# An Observational Study of Post-hip Fracture Outcomes in Older Adults with Type 2 Diabetes

**DOI:** 10.1007/s11606-026-10516-1

**Published:** 2026-06-15

**Authors:** Keity M. Okazaki, Lipi A. Marion, Sherri-Ann M. Burnett-Bowie, Sara J. Cromer, Janinne Ortega-Montiel, Caroline F. Byrne, Elisabetta Patorno, Julie M. Paik, Elaine W. Yu

**Affiliations:** 1 Endocrine Unit, Department of Medicine, Massachusetts General Hospital, Boston, MA, USA; 2 Division of Pharmacoepidemiology and Pharmacoeconomics, Department of Medicine, Brigham and Women’s Hospital, Boston, MA, USA; 3 Division of Internal Medicine and Health Services Research, David Geffen School of Medicine at UCLA, Los Angeles, CA, USA; 4 New England Geriatric Research Education and Clinical Center, VA Boston Healthcare System, Boston, MA, USA

**Keywords:** disparities, osteoporosis, diabetes

## Abstract

**BACKGROUND:**

Hip fracture is a global public health concern, with over 10 million cases worldwide in 2019. Post-hip fracture outcomes, including mortality, vary by type 2 diabetes (T2D) co-diagnosis and patient demographics.

**OBJECTIVE:**

To examine post-hip fracture outcomes and identify factors modifying mortality differences in a racially and ethnically diverse population of older United States (US) adults with T2D.

**DESIGN:**

Retrospective cohort study using Medicare fee-for-service data from 2014 to 2020.

**PARTICIPANTS:**

Men and women aged > 65 years, with T2D diagnosis who had experienced a non-traumatic hip fracture.

**MAIN MEASURE(S):**

We evaluated associations between race and ethnicity and one-year mortality, dual energy x-ray absorptiometry (DXA) testing, osteoporosis treatment, and incident destitution post-hip fracture using multivariable Cox proportional hazards models.

**KEY RESULTS:**

Among 159,699 older adults with T2D, one-year post-hip fracture mortality was 30.4%. In age- and sex-adjusted models, mortality was higher in Black individuals (HR 1.26 [95% CI, 1.22–1.31) and lower in Asian and Hispanic individuals (HR 0.78 [95% CI, 0.73–0.83] and 0.91 [95% CI,0.86–0.96], respectively) compared with White individuals. Elevated mortality in Black individuals persisted across subgroups stratified by sex, age, region, BMI, and frailty, but not among those with advanced chronic kidney disease (CKD stages 4–5; HR 1.01 [0.93–1.09]) or low socioeconomic status (SES; Medicaid eligibility; HR 1.00 [0.88–1.14]). Models adjusting for important demographic and clinical covariates mitigated mortality differences between Black and White individuals. DXA testing and osteoporosis treatment initiation rates were low overall (7.3% and 5.6%, respectively). Black individuals had lower DXA testing (HR 0.51 [0.45–0.56]) and treatment (HR 0.58 [0.51–0.65]) rates, and higher incident destitution (HR 1.41 [1.29–1.55]) than White individuals in all models.

**CONCLUSIONS:**

Overall mortality was high and guideline-concordant care was low in the first year post-hip fracture in Medicare-insured older adults with T2D. Furthermore, racial and ethnic differences in post-hip fracture mortality and management exist.

## INTRODUCTION

Over half of United States (US) adults ≥ 65 years have osteoporosis or low bone mineral density.^1^ Osteoporotic fractures cause significant morbidity and mortality, with hip fracture being associated with ~ 25% one-year mortality and 33% risk of loss of independent living.^2,3^ Despite lower overall fracture incidence, Black individuals have higher mortality and destitution post-hip fracture,^4^ potentially reflecting broader sociodemographic and institutional inequities. Despite evidence-based recommendations for osteoporosis management post-hip fracture,^5^ guideline-concordant care is universally underutilized,^6–8^ with disproportionately lower screening and treatment of Black individuals, with one study showing that within a population where universal screening was recommended, only 19.4% of those screened were Black women compared to 80.6% for White women.^9–12^

Type 2 diabetes (T2D), affecting 29% of US adults ≥ 65 years,^13,14^ increases the risk of hip and nonvertebral fractures^15–19^ and is associated with worse post-fracture outcomes such as higher mortality risk, longer hospital stays, and higher readmission rates.^20–22^ Greater comorbidity burden, including T2D, may contribute to higher post-fracture mortality among Black patients.^23^ However, it remains unclear whether there are post-hip fracture mortality, screening, or treatment differences related to race or ethnicity in a cohort uniformly affected by T2D. Data are especially limited in Asian, Hispanic, and Native American individuals and in men; few large-scale studies have examined mortality, refracture, and osteoporosis care outcomes in cohorts with broad generalizability to the US.

To address these gaps, we conducted a retrospective cohort study of Medicare beneficiaries with T2D who sustained low-trauma hip fracture to evaluate the association between race and ethnicity and post-fracture mortality, refracture, destitution, and quality-of-care metrics, and to investigate potential modifiers of observed differences.

## METHODS

### Data Source, Primary Study Cohort and Exposure Definition

We examined Medicare fee-for-service claims data from Parts A (inpatient), B (outpatient), and D (prescription benefits) from January 2014 through December 2020. We included individuals aged > 65 years with ≥ 365 days of continuous enrollment before cohort entry, a diagnosis of T2D (identified using International Classification of Disease [ICD] 9/10 codes), and encounter for hip fracture (see Supplemental Table 1 for ICD 9/10 codes). The cohort entry date was the hip fracture date (Supplemental Figure 1). We excluded individuals with concurrently billed trauma codes within 15 days before or on the date of the first hip fracture diagnosis using a modified version of a validated algorithm.^4^ Participants missing key demographic variables (age, sex, race, ethnicity) and with conditions predisposing to pathologic fracture (malignancy with potential bone metastasis or Paget’s disease) were also excluded. Race and ethnicity, the primary exposure variables, were defined in the Medicare dataset using self-reported or algorithmically derived data (based on last name and region) and included five mutually exclusive categories: Asian, Black, Hispanic, Native American, and White.^24,25^ Algorithmic classification accuracy is high for White and Black individuals, but lower for Asian and Hispanic individuals;^26^ nonetheless, this variable has been widely used to study health differences in population-based research.^4,24,27–29^ The study was approved by the Mass General Brigham institutional review board; a data use agreement was in place.

### Outcomes Assessment and Subcohort Creation

**Figure 1:**
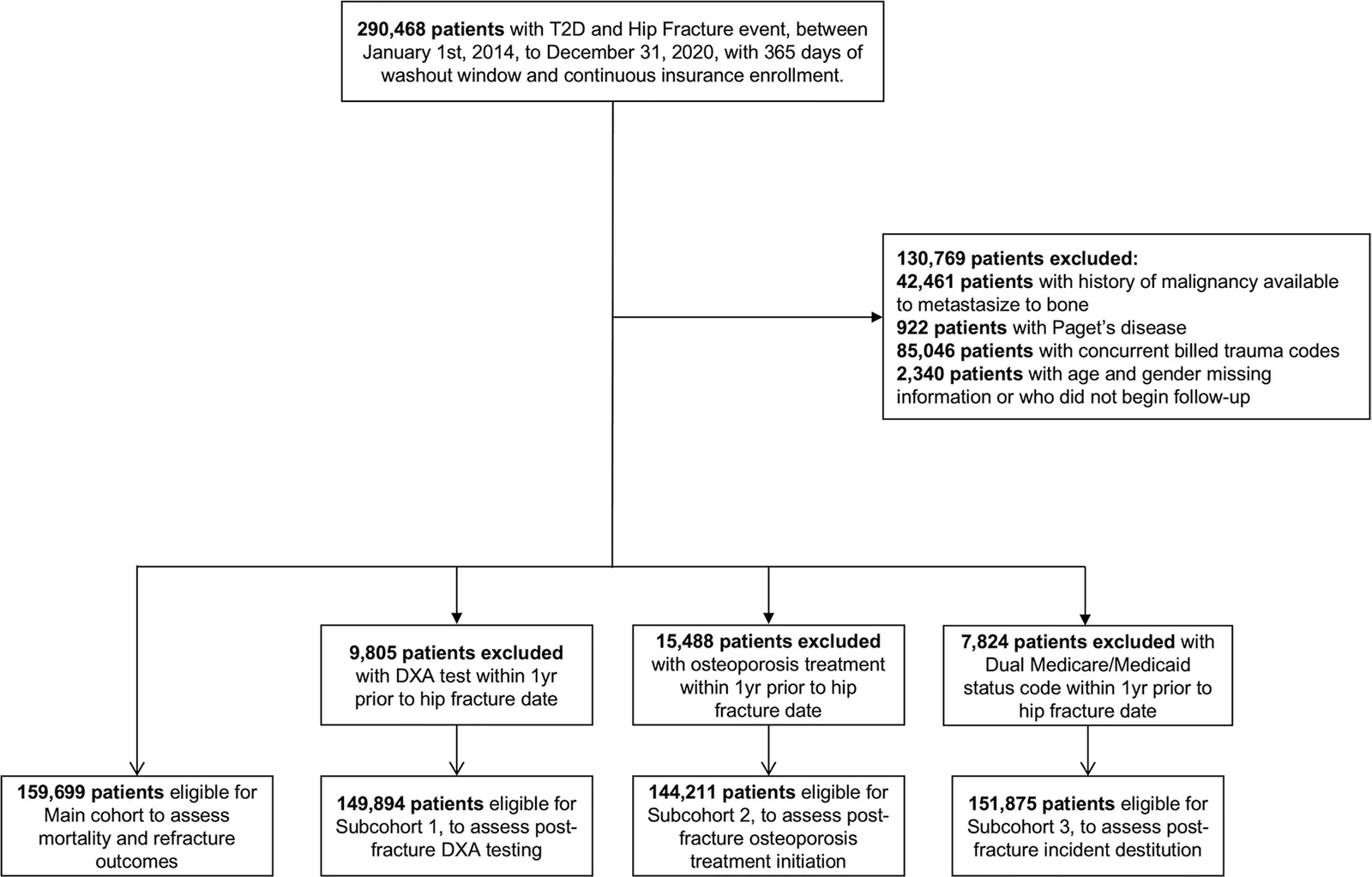
Flowchart of the study main cohort and subcohorts. Abbreviations: T2D, type 2 diabetes; DXA, dual-energy X-ray absorptiometry; yr, year.

The primary outcome was time to death post-hip fracture. The morbidity secondary outcome included time to recurrent fragility fracture, defined as the first atraumatic pelvic, hip, femur, humerus, radius, or ulnar fracture occurring ≥ 90 days from the last claim associated with the initial hip fracture (Supplemental Table 2).^4^ The quality-of-care secondary outcomes were time to dual energy x-ray absorptiometry (DXA) testing, initiation of new osteoporotic therapy (bisphosphonates, denosumab, estrogen-receptor modulators, parathyroid hormone [PTH]-analogs, romosozumab), and incident destitution (defined as new dual eligibility for Medicare and Medicaid) (Supplemental Table 2).^4^ Post-hip fracture DXA testing and osteoporosis treatment initiation were chosen as quality-of-care outcomes based on Healthcare Effectiveness Data and Information Set (HEDIS) standardized performance metrics, which track the percentage of women aged 67–85 years receiving DXA testing or osteoporosis treatment within 180 days post-fracture.^30^ All outcomes were assessed within one year post-hip fracture. All-cause mortality and recurrent fracture analyses were examined in the main cohort. For the remaining secondary outcomes, we created subcohorts restricted to those without recent DXA testing (subcohort 1), not previously on osteoporosis medication (subcohort 2), or not meeting dual Medicare/Medicaid eligibility criteria (subcohort 3), each assessed within 365 days before cohort entry (see Fig. 1).

### Covariates

Baseline covariates were assessed during the 365 days before cohort entry. Demographic variables included age, sex, race and ethnicity, geographic region, and cohort entry year (Supplemental Figure 1). Clinical characteristics included body mass index (BMI) category, smoking status, prior falls,^31,32^ prior fractures, and key metabolic and cardiovascular comorbidities based on ICD-9/10 codes. Claims-based frailty index^33^ and Gagne combined comorbidity index^34^ were used to calculate the combined comorbidity scores and frailty scores. Low socioeconomic status was assessed using a proxy defined by dual Medicare/Medicaid eligibility.^4^ Healthcare utilization measures included medication use (e.g., insulin, osteoporosis agents, corticosteroids), number of unique prescriptions, internist office visits, emergency department visits, DXA testing, and hospitalizations.

Participants were followed until the earliest of 365 days of follow-up, Medicare disenrollment, occurrence of a study outcome, death, or study period end (December 31, 2020).

### Statistical Analysis

Baseline characteristics were compared using ANOVA for continuous variables and Pearson chi-squared test for categorical variables. We used multivariable Cox proportional hazards models to assess racial and ethnic differences in time-to-occurrence of the primary outcome (death), with results reported as hazard ratios (HR) and 95% confidence intervals (CIs) for each race and ethnicity, with White individuals as the reference group. Fine-Gray subdistribution hazard models accounted for death as a competing risk in secondary analyses.^35^ We first ran age- and sex-adjusted models, followed by fully-adjusted models incorporating baseline health status, comorbidities, medications, healthcare utilization, SES, and cohort entry year (see Table 2 footnote for full list of adjustments). Full adjustment was conducted, hypothesizing that persistent differences would indicate unmeasured variables; this approach more effectively clarifies the potential factors underlying observed differences. We calculated age-standardized one-year incidence rates (IRs) per 1,000 person-years by race and ethnicity for each outcome. Kaplan–Meier curves illustrated cumulative mortality by race and ethnicity.

Subgroup analyses evaluated the impact of demographic and comorbid conditions on racial differences in one-year mortality, stratifying by age, sex, geographic region, low SES status, smoking, frailty score, heart failure, chronic obstructive pulmonary disease (COPD), chronic kidney disease (CKD) stage, end-stage kidney disease (ESKD), and insulin use. Age-stratified subgroup analyses were adjusted for sex, sex-stratified analyses were adjusted for age, and all others for both age and sex.

All analyses were performed with Aetion^®^ Substantiate (2025) with R (version 5.44.2) integration and STATA, version 18 (STATACorp, College Station, TX).

## RESULTS

### Baseline Characteristics

Among 159,699 Medicare beneficiaries with T2D and incident hip fracture, mean age was 81.8 (SD 8.0) years, 71.4% were women, and 23.7% had moderate/severe frailty (Table 1). Black and Native American individuals sustained hip fractures at younger ages, with 11.5% and 13.6% of fractures, respectively, occurring between 66–69 years of age, compared with 7.6% in White individuals. Black individuals had more chronic comorbidities than other racial and ethnic groups at baseline, including higher prevalence of advanced kidney disease compared with White individuals (CKD stages 4–5: 16.4% vs 10.1% and ESKD: 18.5% vs. 5.6%). Asian (13.4%), Hispanic (14.2%), and Native American (13.3%) individuals also had higher ESKD prevalence compared with White individuals. High frailty scores were more frequent among Black and Hispanic individuals, and low SES was more common in Black (7.2%), Hispanic (6.1%), and Native American (6.1%) individuals than among White and Asian individuals (4.8% and 2.6%, respectively).

Health care utilization in the year preceding hip fracture was lower in Black individuals, compared to White individuals, with fewer internist office visits and DXA testing (4% vs. 6.4% respectively) but greater utilization of emergency care. Non-hip fracture rates in the year prior to hip fracture were similar between the different groups (range 1.8–2.8%). Osteoporosis diagnosis prior to hip fracture was numerically higher in Asian, Hispanic, and Native American individuals (38.0%, 33.7%, and 31.0%, respectively), and lower in Black (22.1%) compared with White individuals (28.7%). Osteoporosis medication use before fracture in this cohort was highest in Asian (21.3%) and Hispanic (13.2%) individuals, lower in White (9.2%) individuals and Native American (9.9%) individuals, and lowest in Black (5.7%) individuals.

### One-year Outcomes Post-hip Fracture

**Figure 2:**
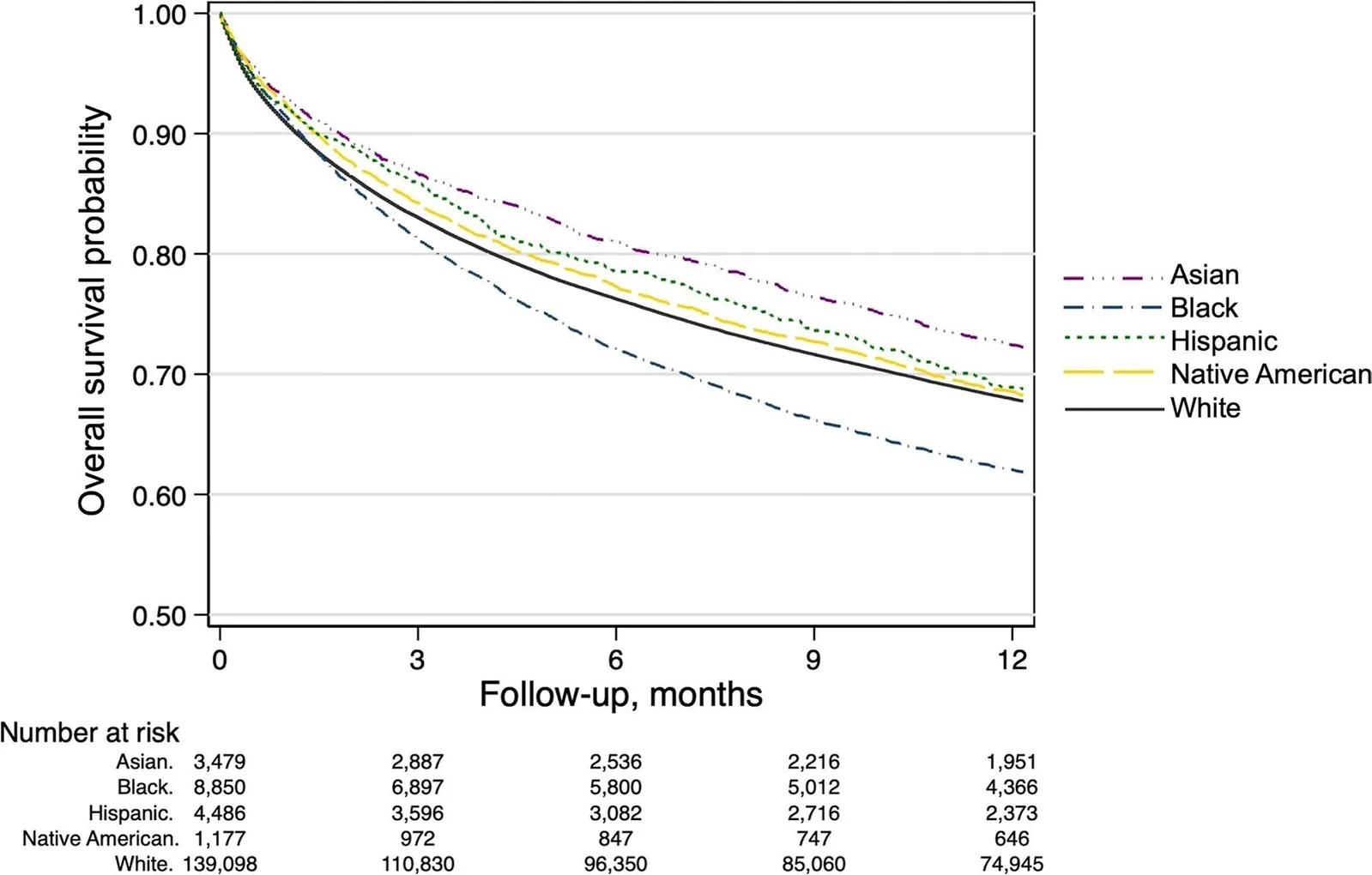
Kaplan–Meier survival curves for 12-month post-hip fracture mortality by race and ethnicity.

Overall, one-year mortality was 30.4%. In age- and sex-adjusted models, Black individuals had higher one-year mortality compared with White individuals (527.8 vs. 428.6 deaths per 1,000 person-years; HR 1.26 [95% CI, 1.22–1.31]; Fig. 2 and Table 2). After further adjustment for clinical covariates, mortality rates were similar in Black and White individuals (HR 1.02 [95% CI, 0.98–1.05]). In contrast, Asian and Hispanic individuals had lower one-year mortality compared with White individuals in both age- and sex-adjusted and fully-adjusted models.

Within one-year post-hip fracture, 3.8% of individuals experienced another fracture. Black individuals had lower refracture rates than White individuals (2.9% vs. 3.9%; HR 0.79 [95% CI, 0.69–0.92] in fully-adjusted model; Table 2). No differences in refracture rate were found in other groups.

Among individuals without recent DXA, one-year post-hip fracture DXA testing was low at 7.3%. Black and Hispanic individuals had significantly lower post-hip fracture DXA testing rates than White individuals (4.3% and 6.0% vs. 7.5%, respectively; Table 2) in both age- and sex-adjusted and fully-adjusted models. Asian individuals had similar rates of testing compared with White individuals in age- and sex-adjusted models, but lower rates after full adjustment (HR 0.83 [95% CI, 0.73–0.95]).

Among individuals not previously taking medication for osteoporosis, osteoporosis treatment initiation within one-year post-fracture was also low at 5.6%. After age- and sex-adjustment, Black individuals were 42% less likely to receive treatment (HR 0.58 [95% CI, 0.51–0.65]), while Asian and Hispanic individuals had higher treatment rates compared with White individuals (Table 2). Differential treatment rates were partially attenuated in fully-adjusted models but remained significant.

Among those not previously eligible for Medicaid, incident destitution occurred in 5.7% of individuals within one-year. Compared with White individuals, new dual Medicare/Medicaid eligibility was higher in Black (HR 1.41 [95% CI, 1.29–1.55]) and Native American (HR 1.36 [95% CI, 1.07–1.73]) individuals and lower in Asian individuals (HR 0.43 [95% CI, 0.34–0.55]) in age- and sex-adjusted, with similar results in fully-adjusted models (Table 2).

### One-year Post-fracture Mortality, Stratified by Clinical Subgroups

**Figure 3:**
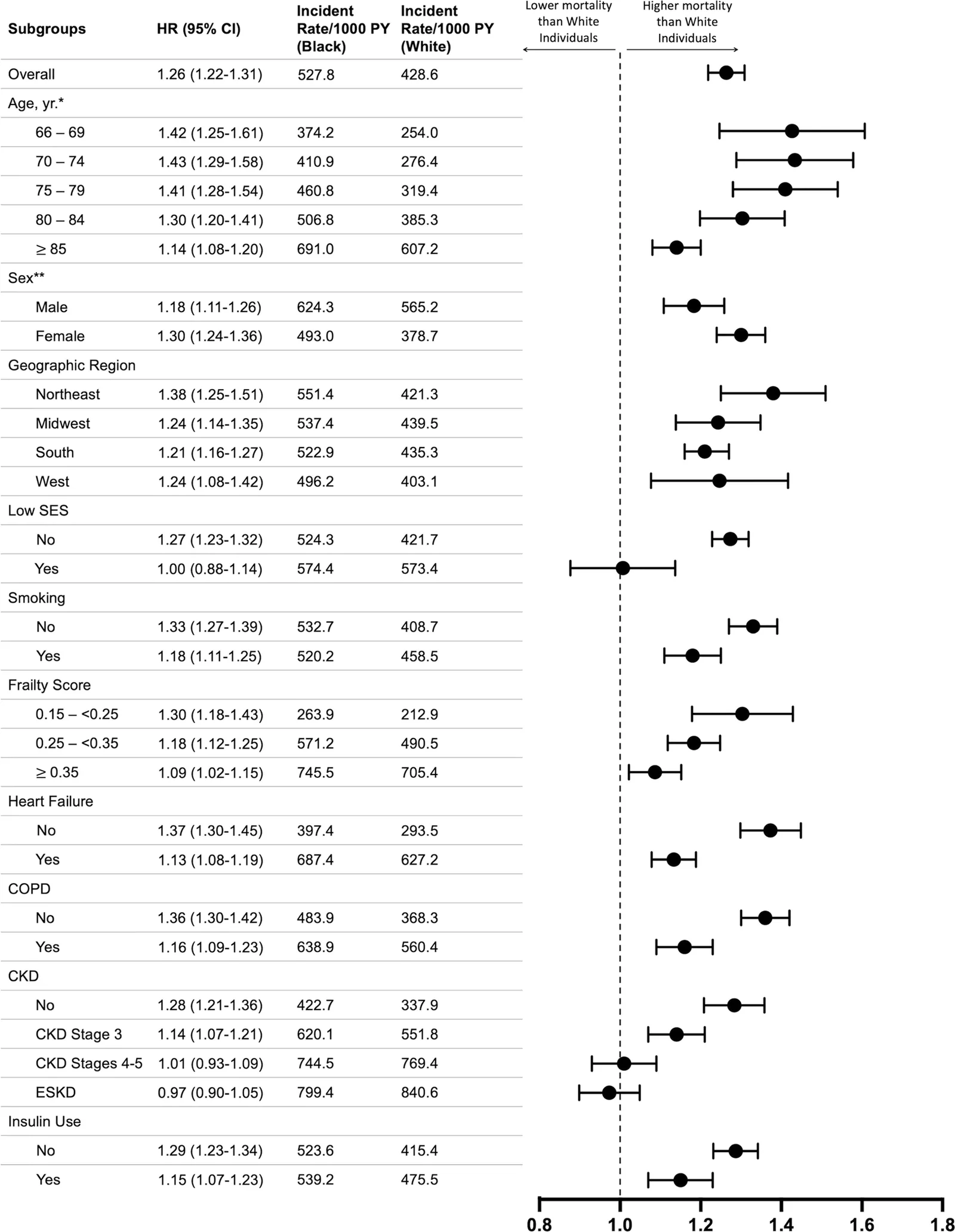
Differences in post-fracture mortality between Black and White individuals in strata of demographic, socioeconomic, and clinical risk factors. Abbreviations: yr, year; SES, socioeconomic status; BMI, body mass index; COPD, chronic obstructive pulmonary disease; CKD, chronic kidney disease; ESKD, end-stage kidney disease. *Age-stratified analysis adjusted for sex only, **Sex-stratified analysis adjusted for age only; other analyses adjusted for both sex and age.

Black individuals had higher mortality than White individuals across all ages after sex-adjustment (Fig. 3), although racial differences were most prominent for younger ages (~ 40% increased mortality risk among ages 66–79) and attenuated at older ages (≥ 85 years; 14% increased mortality risk). Mortality disparities persisted across frailty subgroups, with the largest relative difference observed among less frail individuals (30% higher mortality for Black compared with White individuals). Geographic region did not influence results. Among adults with low baseline SES, mortality did not differ between Black individuals and White individuals (HR 1.00 [0.88–1.14]). Black individuals with fewer comorbidities (heart failure, COPD, or CKD) had greater mortality disparities compared with White individuals in stratified analyses (Fig. 3). Notably, the presence of either CKD stages 4–5 or ESKD prior to hip fracture eliminated differences in post-fracture mortality between Black and White adults in age- and sex-adjusted models (HR 1.01 [95%CI, 0.93–1.09] and HR 0.97 [95% CI, 0.90–1.05], respectively; overall interaction p-value for both < 0.001).

Asian individuals exhibited lower mortality than White individuals independent of sex (Supplemental Table 4). Mortality differences between Asian and White individuals were most prominent in those aged ≥ 80 years (HR 0.70 [95% CI, 0.59–0.82] for ages 80 to 84; HR 0.76 [95% CI, 0.70–0.83] for ages ≥ 85). Among Hispanic individuals, lower post-fracture mortality compared with White individuals was seen in subgroups aged ≥ 85 years, those with low baseline SES, and those with COPD.

## DISCUSSION

In this Medicare claims analysis of older adults with T2D and hip fracture, we found higher age- and sex-adjusted mortality and destitution rates, and lower rates of refracture, DXA testing, and osteoporosis treatment initiation one-year post-hip fracture among Black individuals compared with White individuals. Mortality differences between Black and White individuals were mitigated after adjustment for demographic and clinical covariates, although disparities in refracture, DXA testing, and osteoporosis treatment persisted. Asian and Hispanic individuals experienced lower mortality and higher osteoporosis treatment rates compared with White individuals; Hispanic individuals also had lower DXA testing, while Asian individuals had lower incident destitution. Native American individuals had higher post-fracture destitution rates compared with White individuals. These findings highlight important and previously uncharacterized differences in post-hip fracture outcomes across racial and ethnic groups in older adults with T2D.

Our results extend prior work reporting worse post-hip fracture outcomes in Black compared with White patients,^36,37^ including higher mortality and destitution rates.^4,38,39^ Minoritized US racial and ethnic groups are disproportionately affected by T2D, with the highest prevalence among Native American adults (13.6%), followed by non-Hispanic Black adults (12.1%).^40^ As T2D is associated with higher post-hip fracture mortality, higher baseline T2D prevalence has been cited as a contributing factor to racial mortality differences post-hip fracture.^23^ Our study, however, demonstrated persistent racial differences in age- and sex-adjusted post-hip fracture outcomes in a cohort with T2D, suggesting that factors other than T2D are contributory.

After adjustment for all available clinical and sociodemographic factors, post-fracture mortality no longer significantly differed between Black and White individuals, indicating that the variables included in the adjustment may explain much of the observed difference. Stratified analyses also revealed that racial disparities in mortality between Black and White individuals were most pronounced for younger patients as well as those with better health status or higher socioeconomic standing, suggesting that additional factors likely contribute to this disparity. In contrast, among patients with advanced kidney disease and other comorbidities, increased frailty, or lower socioeconomic standing, mortality rates were more similar between Black and White adults. It is possible that the presence of these social and medical conditions has an outsized impact on increasing absolute mortality that reduces the relative mortality gap between Black and White individuals after hip fracture. Nevertheless, a higher baseline prevalence of these chronic health conditions and lower socioeconomic standing in Black adults may contribute to the overall observed higher mortality compared to White individuals after hip fracture. For instance, Black individuals had higher baseline rates of advanced CKD (34.9% vs. 15.8% in White individuals), heart failure, frailty, and low SES (7.2% vs 4.8% in Black individuals compared with White individuals), factors that have been associated with increased mortality post-hip fracture.^39,41–43^ Structural racism, chronic stress, and social determinants of health have been studied as potential drivers of racial and ethnic disparities in mortality between Black and White individuals.^44–46^ Additional studies are needed to clarify how these mechanisms may contribute to disparities in post-hip fracture mortality.

In contrast, Asian, Hispanic, and Native American individuals—despite higher baseline ESKD rates—did not exhibit a similarly increased mortality. Instead, Asian and Hispanic individuals experienced lower post-hip fracture mortality, consistent with prior studies in older women post-hip fracture.^39^ These findings may reflect protective environmental and behavioral factors described as the “healthy immigrant effect”.^47,48^ Higher baseline (both) and post-fracture (Asian individuals only) osteoporosis treatment rates may also contribute, as osteoporosis therapy has been associated with reduced post-fracture mortality.^49,50^

Low SES is a well-established risk factor for worse outcomes after hip fracture, mediated through disparities in post-acute care, nutrition, environmental exposures, and comorbidity burden.^51,52^ Our SES proxy of dual Medicare/Medicaid eligibility captures income and household size but may systematically underestimate the true extent of socioeconomic hardship for Black individuals, particularly relative to Asian individuals, due to geographic clustering of racial groups and state-level variability in eligibility thresholds.^53,54^ Nevertheless, we found that post-hip fracture incident destitution was higher among Black and Native American compared with White individuals, extending previous findings in older Black women^4^ to include older Black men and Native American adults.

Black patients sustained hip fractures at younger ages and demonstrated greater relative mortality differences at younger ages. It is possible that the use of racial and ethnic adjustment factors in US-FRAX could be contributing to undertreatment of Black individuals at high risk;^55^ indeed we found markedly lower baseline osteoporosis treatment rates in Black individuals, which have also been documented in prior studies.^9^ The smaller mortality difference observed among Black individuals aged ≥ 85 years compared with White individuals may reflect survivorship bias in this group.

Black individuals were less likely to experience a second fracture compared with White individuals. To our knowledge, this is the first study to examine subsequent fracture rates by race and ethnicity, and our findings are concordant with prior studies showing higher incidence of initial fractures in White individuals compared with other races and ethnicities.^56,57^ However, we found no significant differences in refracture rates between Asian, Hispanic, and Native American individuals compared with White individuals.

Black and Hispanic individuals were also less likely to undergo DXA testing within one-year post-hip fracture, consistent with prior studies.^57–59^ Additionally, Black individuals had lower rates of osteoporosis treatment initiation post-hip fracture. Although limited, existing studies examining post-fracture pharmacotherapy by race and ethnicity have identified Black race as a factor associated with reduced likelihood of receiving DXA testing or osteoporosis treatment within six months of a hip fracture.^60^ Our study expands these observations to a larger, more racially diverse cohort, while specifically focusing on patients with T2D, a condition known to increase skeletal fragility.

Lastly, guideline-based prevention and treatment of osteoporotic fractures was very low, both before and after hip fracture occurrence. In the year prior to fracture, only 6.2% of individuals underwent DXA testing, despite 71.4% of the cohort being women aged > 65 years with T2D, a high-risk population meeting criteria for DXA testing per the US Preventive Services Task Force.^5^ These care gaps likely contributed to baseline underdiagnosis and undertreatment of osteoporosis in our cohort. Similarly, post-hip fracture osteoporosis care was limited, with only 7.3% of individuals without recent screening receiving DXA testing and only 5.6% of individuals previously untreated receiving osteoporosis treatment in the year after fracture, despite hip fracture being a strong indication for therapy. Our findings underscore the underutilization of effective osteoporosis therapies despite clear evidence of benefit,^61^ universal post-hip fracture treatment and assessment recommendations,^62^ and established HEDIS performance metrics.^30,63–65^

This study has limitations, including a small Native American cohort, exclusion of vertebral fractures given challenges with accurate coding for temporality, potential underestimation of SES disparities, limitations in assessment of social determinants of health, possible misclassification of race in Medicare data, and missing data. ^23,47,48^ Nonetheless, its strengths include use of a large, nationally representative cohort that, except for Native American individuals, enabled robust subgroup analyses, and inclusion of men, who are underrepresented in osteoporosis research. Importantly, while previous studies have described racial and ethnic differences post-hip fracture, our study advances knowledge by investigating factors that modify these disparities in a T2D population.

In summary, older Black US adults with T2D experience higher post-hip fracture mortality compared with older White adults, potentially due to baseline risk factors and disproportionately low rates of appropriate post-hip fracture care. Our findings underscore the need for clinicians and healthcare systems to increase efforts focused on fragility fracture prevention and treatment in all racial and ethnic groups, given the noted poor post-hip fracture outcomes. Finally, our findings characterize the extent of negative post-hip fracture outcomes that older Black individuals with T2D experience, reinforcing the urgent need to improve equitable osteoporosis care delivery.

## Supplementary Material

Supplement

**Supplementary Information** The online version contains supplementary material available at https://doi.org/10.1007/s11606-026-10516-1.

## Figures and Tables

**Table 1:** Baseline Demographics, Overall and Stratified by Race and Ethnicity

Population Characteristics	All Patients	Asian	Black	Hispanic	Native American	White

Number (%) or Mean (SD)	159,699 (100%)	3,479 (2.2%)	8,850 (5.5%)	4,486 (2.8%)	1,177 (0.7%)	139,098 (87.1%)
Age, yr	81.8 (8.0)	83.1 (7.9)	80.9 (8.6)	83.2 (8.3)	79.0 (7.6)	81.9 (8.0)
Age categories						
66–69 yr	12,691 (7.9%)	217 (6.2%)	1,018 (11.5%)	326 (7.3%)	160 (13.6%)	10,574 (7.6%)
70–74 yr	21,509 (13.5%)	373 (10.7%)	1,357 (15.3%)	508 (11.3%)	187 (15.9%)	18,662 (13.4%)
75–79 yr	27,424 (17.2%)	520 (14.9%)	1,534 (17.3%)	622 (13.9%)	261 (22.2%)	24,057 (17.3%)
80–84 yr	33,357 (20.9%)	724 (20.8%)	1,738 (19.6%)	806 (18.0%)	271 (23.0%)	29,288 (21.1%)
≥ 85 yr	64,718 (40.5%)	1,645 (47.3%)	3,203 (36.2%)	2,224 (49.6%)	298 (25.3%)	56,517 (40.6%)
Female	114,067 (71.4%)	2,552 (73.4%)	6,395 (72.3%)	3,207 (71.5%)	899 (76.4%)	99,420 (71.5%)
Geographic region						
Northeast	31,295 (19.6%)	646 (18.6%)	1,333 (15.1%)	492 (11.0%)	23 (2.0%)	28,210 (20.3%)
South	67,746 (42.4%)	662 (19.0%)	5,320 (60.1%)	2,096 (46.7%)	374 (31.8%)	58,610 (42.1%)
Midwest	33,922 (21.2%)	188 (5.4%)	1,575 (17.8%)	256 (5.7%)	141 (12.0%)	31,419 (22.6%)
West	26,736 (16.7%)	1,983 (57.0%)	622 (7.0%)	1,642 (36.6%)	639 (54.3%)	20,859 (15.0%)
Low SES	7,921 (5.0%)	89 (2.6%)	637 (7.2%)	273 (6.1%)	72 (6.1%)	6,745 (4.8%)
BMI categories						
< 25 kg/m^2^	17,061 (10.7%)	564 (16.2%)	1,316 (14.9%)	569 (12.7%)	104 (8.8%)	14,180 (10.2%)
25–29.9 kg/m^2^	14,558 (9.1%)	254 (7.3%)	739 (8.4%)	498 (11.1%)	83 (7.1%)	12,733 (9.2%)
30–39.9 kg/m^2^	15,895 (10.0%)	122 (3.5%)	729 (8.2%)	440 (9.8%)	108 (9.2%)	14,253 (10.2%)
≥ 40 kg/m^2^	9,596 (6.0%)	57 (1.6%)	553 (6.2%)	224 (5.0%)	60 (5.1%)	8,568 (6.2%)
Smoking	62,942 (39.4%)	643 (18.5%)	3,472 (39.2%)	1,191 (26.5%)	469 (39.8%)	56,353 (40.5%)
Frailty scores						
< 0.15 (robust)	2,494 (1.6%)	97 (2.8%)	94 (1.1%)	56 (1.2%)	20 (1.7%)	2,148 (1.5%)
0.15-< 0.25 (pre-frailty)	50,667 (31.7%)	1,373 (39.5%)	2,204 (24.9%)	1,207 (26.9%)	433 (36.8%)	44,432 (31.9%)
0.25-< 0.35 (mild frailty)	68,625 (43.0%)	1,399 (40.2%)	3,876 (43.8%)	1,886 (42.0%)	486 (41.3%)	59,941 (43.1%)
≥ 0.35 (moderate frailty and above)	37,913 (23.7%)	610 (17.5%)	2,676 (30.2%)	1,337 (29.8%)	238 (20.2%)	32,577 (23.4%)
Falls	39,856 (25.0%)	630 (18.1%)	2,046 (23.1%)	1,003 (22.4%)	369 (31.4%)	35,250 (25.3%)
Comorbidities						
Combined Comorbidity Score	3.4 (2.99)	3.0 (3.04)	4.3 (3.34)	3.7 (3.08)	3.5 (3.05)	3.3 (2.95)
Diabetic retinopathy	20,972 (13.1%)	502 (14.4%)	1,221 (13.8%)	773 (17.2%)	223 (18.9%)	17,828 (12.8%)
Diabetic neuropathy	43,701 (27.4%)	795 (22.9%)	2,603 (29.4%)	1,513 (33.7%)	341 (29.0%)	37,741 (27.1%)
Diabetic nephropathy	37,479 (23.5%)	1,084 (31.2%)	2,864 (32.4%)	1,386 (30.9%)	384 (32.6%)	30,989 (22.3%)
Diabetic foot ulcer	15,505 (9.7%)	184 (5.3%)	1,028 (11.6%)	441 (9.8%)	136 (11.6%)	13,498 (9.7%)
Heart failure	70,525 (44.2%)	1,324 (38.1%)	4,267 (48.2%)	1,936 (43.2%)	485 (41.2%)	61,510 (44.2%)
COPD	52,079 (32.6%)	752 (21.6%)	2,628 (29.7%)	1,330 (29.6%)	379 (32.2%)	46,373 (33.3%)
CKD Stage 3	42,988 (26.9%)	831 (23.9%)	2,425 (27.4%)	1,054 (23.5%)	294 (25.0%)	37,732 (27.1%)
CKD Stages 4–5	16,985 (10.6%)	445 (12.8%)	1,448 (16.4%)	575 (12.8%)	151 (12.8%)	14,033 (10.1%)
End-stage kidney disease*	11,060 (6.9%)	465 (13.4%)	1,639 (18.5%)	636 (14.2%)	157 (13.3%)	7,839 (5.6%)
Osteoporosis	45,728 (28.6%)	1,322 (38.0%)	1,953 (22.1%)	1,512 (33.7%)	365 (31.0%)	39,859 (28.7%)
Fracture in prior year	3,861 (2.4%)	61 (1.8%)	175 (2.0%)	108 (2.4%)	33 (2.8%)	3,427 (2.5%)
Medications						
Number of unique generic medications	13.5 (7.0)	13.8 (7.8)	13.0 (6.9)	14.5 (7.6)	13.5 (7.5)	13.5 (6.9)
Insulin	35,712 (22.4%)	714 (20.5%)	2,319 (26.2%)	1,297 (28.9%)	400 (34.0%)	30,357 (21.8%)
Osteoporosis medications**	15,069 (9.4%)	741 (21.3%)	508 (5.7%)	592 (13.2%)	116 (9.9%)	12,784 (9.2%)
Oral Corticosteroids	33,368 (20.9%)	524 (15.1%)	1,474 (16.7%)	690 (15.4%)	227 (19.3%)	30,041 (21.6%)
Health system utilization (prior 365 days)						
DXA testing	9,992 (6.3%)	233 (6.7%)	356 (4.0%)	300 (6.7%)	53 (4.5%)	8,883 (6.4%)
Internist outpatient visits	125,704 (78.7%)	2,902 (83.4%)	6,504 (73.5%)	3,464 (77.2%)	906 (77.0%)	109,844 (79.0%)

Abbreviations: *SES* socioeconomic status, *BMI* body mass index (calculated as weight in kilograms divided by height in meters squared), *COPD* chronic obstructive pulmonary disease, *CKD* chronic kidney disease, *DXA* dual-energy X-ray absorptiometry

* Includes dialysis,

** Excludes calcium and vitamin D

**Table 2:** Hazard Ratios for one-year Post-Fracture Mortality, Refracture, DXA Testing, Osteoporosis Medication Initiation, and Destitution Rates by Race and Ethnicity

Outcome	Asian HR (95% CI)	Black HR (95% CI)	Hispanic HR (95% CI)	Native American HR (95% CI)	White

**Total Subcohort (N)**	3,479	8,850	4,486	1,177	139,098
**Mortality N (%)**	896 (25.7)	3,168 (35.8)	1,318 (29.4)	344 (29.2)	42,209 (30.3)
Unadjusted	0.82 (0.76, 0.87)	1.20 (1.16, 1.25)	0.96 (0.91, 1.02)	0.94 (0.84, 1.04)	REF
Age and Sex adjusted	0.78 (0.73, 0.83)	1.26 (1.22, 1.31)	0.91 (0.86, 0.96)	1.06 (0.96, 1.18)	REF
Fully adjusted*	0.87 (0.81, 0.93)	1.02 (0.98, 1.05)	0.87 (0.82, 0.92)	1.00 (0.90, 1.11)	REF
**Total Subcohort (N)**	3,479	8,850	4,486	1,177	139,098
**Refracture N (%)**	114 (3.3)	255 (2.9)	170 (3.8)	48 (4.1)	5,460 (3.9)
Unadjusted	0.82 (0.67, 1.00)	0.76 (0.66, 0.87)	0.95 (0.80, 1.13)	1.06 (0.77, 1.44)	REF
Age and Sex adjusted	0.82 (0.67, 1.00)	0.76 (0.66, 0.87)	0.95 (0.80, 1.13)	1.04 (0.76, 1.41)	REF
Fully adjusted*	0.88 (0.72, 1.09)	0.79 (0.69, 0.92)	0.97 (0.82, 1.16)	1.04 (0.76, 1.43)	REF
**Total Subcohort (N)**	3,248	8,499	4,193	1,125	130,393
**DXA Testing N (%)**	258 (7.9)	369 (4.3)	250 (6.0)	96 (8.5)	9,811 (7.5)
Unadjusted	1.02 (0.89, 1.15)	0.54 (0.48, 0.60)	0.77 (0.67, 0.87)	1.07 (0.87, 1.32)	REF
Age and Sex adjusted	1.08 (0.95, 1.23)	0.51 (0.45, 0.56)	0.83 (0.73, 0.95)	0.90 (0.72, 1.11)	REF
Fully adjusted*	0.83 (0.73, 0.95)	0.62 (0.56, 0.70)	0.81 (0.71, 0.92)	0.92 (0.74, 1.13)	REF
**Total Subcohort (N)**	2,710	8,329	3,881	1,059	125,976
**Initiation of Osteoporosis treatment N (%)**	221 (8.1)	281 (3.4)	245 (6.3)	90 (8.5)	7,133 (5.7)
Unadjusted	1.44 (1.25, 1.65)	0.60 (0.53, 0.68)	1.11 (0.97, 1.27)	1.34 (1.06, 1.68)	REF
Age and Sex adjusted	1.53 (1.33, 1.76)	0.58 (0.51, 0.65)	1.18 (1.03, 1.35)	1.18 (0.93, 1.48)	REF
Fully adjusted*	1.28 (1.11, 1.48)	0.76 (0.66, 0.86)	1.15 (1.00, 1.32)	1.12 (0.89, 1.42)	REF
**Total Subcohort (N)**	3,393	8,220	4,218	1,105	132,446
**Destitution N (%)**	80 (2.4)	632 (7.7)	212 (5.0)	78 (7.0)	7,599 (5.7)
Unadjusted	0.44 (0.35, 0.56)	1.41 (1.28, 1.54)	0.93 (0.80, 1.09)	1.35 (1.06, 1.71)	REF
Age and Sex adjusted	0.43 (0.34, 0.55)	1.41 (1.29, 1.55)	0.92 (0.79, 1.07)	1.36 (1.07, 1.73)	REF
Fully adjusted*	0.58 (0.46, 0.74)	1.30 (1.18, 1.43)	1.02 (0.87, 1.18)	1.58 (1.24, 2.01)	REF

Abbreviations: *DXA* dual-energy X-ray absorptiometry.

* Fully-adjusted model adjusted for age, sex, geographic region, cohort entry year, low socioeconomic status, body mass index, smoking, falls, combined comorbidity score, diabetic retinopathy, diabetic neuropathy, diabetic foot ulcer, chronic kidney disease stage 3, chronic kidney disease stages 4–5, end stage kidney disease, frailty score, hypertension, myocardial infarction, ischemic stroke, heart failure, atrial fibrillation, hyperlipidemia, hyperthyroidism, cognitive impairment, depression, osteoporosis treatment, osteoporosis, liver disease, cancer, chronic obstructive pulmonary disease, fracture in prior year, alcohol use disorder, number of generic medications, number of brand medications, antiarrhythmic medication, antidepressant medication, anxiolytic medication, benzodiazepine medication, beta blocker medication, calcium channel blockers medication, corticosteroids medication, insulin use, opioids medication, proton pump inhibitor medication, Sodium-Glucose Cotransporter 2 Inhibitors medication, Thiazolidinediones medication, angiotensin-converting enzyme inhibitors or angiotensin receptor blockers medications, emergency department visits, hospitalization events, internist outpatient visits, dual-energy X-ray absorptiometry testing

## Data Availability

The data used in this study are governed by data use agreements that preclude the authors from sharing patient-level or derived data. The administrative and clinical research databases used in this study are accessible to other researchers by contacting the data providers and executing the appropriate data use agreements or licenses. Research data and any derived datasets cannot be shared outside the terms of these agreements.
