# Supplementary material for: An Observational Study of Post-hip Fracture Outcomes in Older Adults with Type 2 Diabetes
Source: J Gen Intern Med. Author manuscript; Available in PMC 2026 Sep 28. (PMC13618796; doi:10.1007/s11606-026-10516-1)
Supplement: Supplementary file 1 [file NIHMS2209106-supplement-Supplement.docx]

**Supplemental Material – Table of Contents**

**Supplemental Table 1.** International Classification of Disease codes used to identify hip fracture

**Supplemental Figure 1.** Study Design Diagram

**Supplemental Table 2.** International Classification of Disease codes used for outcome assessment

**Supplemental Table 3.** Complete Patient Demographics

**Supplemental Table 4.** Differences in post-fracture mortality between Asian, Black, Hispanic, and Native American individuals versus White individuals in strata of demographic, socioeconomic, and clinical risk factors

**Supplemental Table 1.** International Classification of Disease codes used to identify hip fracture

| **ICD9 Dx** | **Specific code definitions** | **ICD10 Dx** | **Specific code definitions** |
| --- | --- | --- | --- |
| 73314 | Pathologic fracture of neck of femur | S72.001A | Fracture of unspecified part of neck of right femur, initial encounter for closed fracture |
| 82000 | Closed fracture of intracapsular section of neck of femur, unspecified | S72.002A | Fracture of unspecified part of neck of left femur, initial encounter for closed fracture |
| 82001 | Closed fracture of epiphysis (separation) (upper) of neck of femur | S72.009A | Fracture of unspecified part of neck of unspecified femur, initial encounter for closed fracture |
| 82002 | Closed fracture of midcervical section of neck of femur | S72.009C | Fracture of unspecified part of neck of femur unspecified |
| 82003 | Closed fracture of base of neck of femur | S72.011A | Unspecified intracapsular fracture of right femur, initial encounter for closed fracture |
| 82009 | Other closed transcervical fracture of neck of femur | S72.012A | Unspecified intracapsular fracture of left femur, initial encounter for closed fracture |
| 82020 | Closed fracture of trochanteric section of neck of femur | S72.019A | Unspecified intracapsular fracture of unspecified femur, initial encounter for closed fracture |
| 82021 | Closed fracture of intertrochanteric section of neck of femur | [S72.021A](https://www.icd10data.com/ICD10CM/Codes/S00-T88/S70-S79/S72/S72.0-/S72.021A) | Displaced fracture of epiphysis (separation) (upper) of right femur (initial encounter) |
| 82080 | Closed fracture of unspecified part of neck of femur | [S72.022A](https://www.icd10data.com/ICD10CM/Codes/S00-T88/S70-S79/S72/S72.0-/S72.022A) | Displaced fracture of epiphysis (separation) (upper) of left femur (initial encounter) |
| 82100 | Closed fracture of femur, NOS | [S72.023A](https://www.icd10data.com/ICD10CM/Codes/S00-T88/S70-S79/S72/S72.0-/S72.023A) | Displaced fracture of epiphysis (separation) (upper) of unspecified femur (initial encounter) |
|  |  | [S72.024A](https://www.icd10data.com/ICD10CM/Codes/S00-T88/S70-S79/S72/S72.0-/S72.024A) | Nondisplaced fracture of epiphysis (separation) (upper) of right femur (initial encounter) |
|  |  | [S72.025A](https://www.icd10data.com/ICD10CM/Codes/S00-T88/S70-S79/S72/S72.0-/S72.025A) | Nondisplaced fracture of epiphysis (separation) (upper) of left femur (initial encounter) |
|  |  | [S72.026A](https://www.icd10data.com/ICD10CM/Codes/S00-T88/S70-S79/S72/S72.0-/S72.026A) | Nondisplaced fracture of epiphysis (separation) (upper) of unspecified femur (initial encounter) |
|  |  | S72.031A | Displaced midcervical fracture of right femur, initial encounter for closed fracture |
|  |  | S72.032A | Displaced midcervical fracture of left femur, initial encounter for closed fracture |
|  |  | S72.033A | Displaced midcervical fracture of unspecified femur, unspecified |
|  |  | S72.034A | Nondisplaced midcervical fracture of right femur, initial encounter for closed fracture |
|  |  | S72.035A | Nondisplaced midcervical fracture of left femur, initial encounter for closed fracture |
|  |  | S72.036A | Nondisplaced midcervical fracture of unspecified femur, initial encounter for closed fracture |
|  |  | S72.041A | Displaced fracture of base of neck of right femur, initial encounter for closed fracture |
|  |  | S72.042A | Displaced fracture of base of neck of left femur, initial encounter for closed fracture |
|  |  | S72.043A | Displaced fracture of base of neck of unspecified femur, initial encounter for closed fracture |
|  |  | S72.044A | Nondisplaced fracture of base of neck of right femur, initial encounter for closed fracture |
|  |  | S72.045A | Nondisplaced fracture of base of neck of left femur, initial encounter for closed fracture |
|  |  | S72.046A | Nondisplaced fracture of base of neck of unspecified femur, initial encounter for closed fracture |
|  |  | S72.051A | Unspecified fracture of head of right femur, initial encounter for closed fracture |
|  |  | S72.052A | Unspecified fracture of head of left femur, initial encounter for closed fracture |
|  |  | S72.059A | Unspecified fracture of head of unspecified femur, initial encounter for closed fracture |
|  |  | S72.061A | Displaced articular fracture of head of right femur, initial encounter for closed fracture |
|  |  | S72.062A | Displaced articular fracture of head of left femur, initial encounter for closed fracture |
|  |  | S72.063A | Displaced articular fracture of head of unspecified femur, initial encounter for closed fracture |
|  |  | S72.064A | Nondisplaced articular fracture of head of right femur, initial encounter for closed fracture |
|  |  | S72.065A | Nondisplaced articular fracture of head of left femur, initial encounter for closed fracture |
|  |  | S72.066A | Nondisplaced articular fracture of head of unspecified femur, initial encounter for closed fracture |
|  |  | S72.091A | Other fracture of head and neck of right femur, initial encounter for closed fracture |
|  |  | S72.092A | Other fracture of head and neck of left femur, initial encounter for closed fracture |
|  |  | S72.099A | Other fracture of head and neck of unspecified femur, initial encounter for closed fracture |
|  |  | S72.101A | Unspecified trochanteric fracture of right femur, initial encounter for closed fracture |
|  |  | S72.102A | Unspecified trochanteric fracture of left femur, initial encounter for closed fracture |
|  |  | S72.109A | Unspecified trochanteric fracture of unspecified femur, initial encounter for closed fracture |
|  |  | S72.111A | Displaced fracture of greater trochanter of right femur, initial encounter for closed fracture |
|  |  | S72.112A | Displaced fracture of greater trochanter of left femur, initial encounter for closed fracture |
|  |  | S72.113A | Displaced fracture of greater trochanter of unspecified femur, initial encounter for closed fracture |
|  |  | S72.114A | Nondisplaced fracture of greater trochanter of right femur, initial encounter for closed fracture |
|  |  | S72.115A | Nondisplaced fracture of greater trochanter of left femur, initial encounter for closed fracture |
|  |  | S72.116A | Nondisplaced fracture of greater trochanter of unspecified femur, initial encounter for closed fracture |
|  |  | S72.121A | Displaced fracture of lesser trochanter of right femur, initial encounter for closed fracture |
|  |  | S72.122A | Displaced fracture of lesser trochanter of left femur, initial encounter for closed fracture |
|  |  | S72.123A | Displaced fracture of lesser trochanter of unspecified femur, initial encounter for closed fracture |
|  |  | S72.124A | Nondisplaced fracture of lesser trochanter of right femur, initial encounter for closed fracture |
|  |  | S72.125A | Nondisplaced fracture of lesser trochanter of left femur, initial encounter for closed fracture |
|  |  | S72.126A | Nondisplaced fracture of lesser trochanter of unspecified femur, initial encounter for closed fracture |
|  |  | S72.131A | Displaced apophyseal fracture of right femur, initial encounter for closed fracture |
|  |  | S72.132A | Displaced apophyseal fracture of left femur, initial encounter for closed fracture |
|  |  | S72.133A | Displaced apophyseal fracture of unspecified femur, initial encounter for closed fracture |
|  |  | S72.134A | Nondisplaced apophyseal fracture of right femur, initial encounter for closed fracture |
|  |  | S72.135A | Nondisplaced apophyseal fracture of left femur, initial encounter for closed fracture |
|  |  | S72.136A | Nondisplaced apophyseal fracture of unspecified femur, initial encounter for closed fracture |
|  |  | S72.141A | Displaced intertrochanteric fracture of right femur, initial encounter for closed fracture |
|  |  | S72.142A | Displaced intertrochanteric fracture of left femur, initial encounter for closed fracture |
|  |  | S72.143A | Displaced intertrochanteric fracture of unspecified femur, initial encounter for closed fracture |
|  |  | S72.144A | Nondisplaced intertrochanteric fracture of right femur, initial encounter for closed fracture |
|  |  | S72.145A | Nondisplaced intertrochanteric fracture of left femur, initial encounter for closed fracture |
|  |  | S72.146A | Nondisplaced intertrochanteric fracture of unspecified femur, initial encounter for closed fracture |
|  |  | S72.8X1A | Other fracture of right femur, initial encounter for closed fracture |
|  |  | S72.8X2A | Other fracture of left femur, initial encounter for closed fracture |
|  |  | S72.8X9A | Other fracture of unspecified femur, initial encounter for closed fracture |
|  |  | S72.91XA | Unspecified fracture of right femur, initial encounter for closed fracture |
|  |  | S72.92XA | Unspecified fracture of left femur, initial encounter for closed fracture |
|  |  | S79.001A | Unspecified physeal fracture of upper end of right femur, initial encounter for closed fracture |
|  |  | S79.002A | Unspecified physeal fracture of upper end of right femur, initial encounter for closed fracture |
|  |  | S79.009A | Unspecified physeal fracture of upper end of left femur, initial encounter for closed fracture |
|  |  | S79.091A | Other physeal fracture of the right upper end of femur, initial encounter for closed fracture |
|  |  | S79.092A | Other physeal fracture of the right upper end of femur, initial encounter for closed fracture |
|  |  | S79.099A | Other physeal fracture of upper end of unspecified femur, initial encounter for closed fracture |
|  |  | M80.051A | Age‐related osteoporosis with current pathological fracture, right femur, initial encounter |
|  |  | M80.052A | Age‐related osteoporosis with current pathological fracture, left femur, initial encounter |
|  |  | M80.059A | Age-related osteoporosis with current pathological fracture, unspecified femur, initial encounter for fracture |
|  |  | M80.851A | Other osteoporosis with current pathological fracture, right femur, initial encounter for fracture |
|  |  | M80.852A | Other osteoporosis with current pathological fracture, left femur, initial encounter for fracture |
|  |  | M80.859A | Other osteoporosis with current pathological fracture, unspecified femur, initial encounter for fracture |
|  |  | M84.451A | Pathological fracture, right femur, initial encounter for fracture |
|  |  | M84.452A | Pathological fracture, left femur, initial encounter for fracture |
|  |  | M84.453A | Pathological fracture, unspecified femur, initial encounter for fracture |
|  |  | M84.459A | Pathological fracture, hip, unspecified, initial encounter for fracture |

Abbreviations: ICD, International Classification of Disease. Dx, diagnosis.

**Supplemental Figure 1.** Study Design Diagram.

^†^Up to 60-day gaps in medical or pharmacy enrollment allowed. ^††^Baseline conditions included: demographics, cohort entry year, lifestyle, diabetes related complications, combined comorbidity score, frailty, score, general comorbidities, other medications, healthcare utilization. ^‡^Earliest outcome of interest, disenrollment, or end of study period.

**Supplemental Table 2.** International Classification of Disease codes used for outcome assessment

| **Primary outcomes** | **Definition codes** | **Setting / Position** |
| --- | --- | --- |
| Death | Vital status - Date of death | Patient attribute |
| Refractures | Incident Pelvis fractures, or  **ICD-9 Dx Codes:** 808.0, 808.1, 808.2, 808.42, 808.44, 808.49, 808.51, 808.52, 808.53, 808.54, 808.8, 808.9, 808.3, 808.4, 808.41, 808.43, 808.5, 808.59.  **ICD-10 Dx Codes:** M84.650A, S32.3xxx, S32.4xxx, S32.5xxx, S32.6xxx, S32.8xxx, S32.9xxx, M84.454A.  **HCPCS Procedure Code:** 27193, 27194, 27218, 27220, 27222, 27226, 27227, G0412, G0413, G0415, 27215, 27216, 27217, 27228, 27254, G0414.  Incident Hip and femur fractures, or  **ICD-9 Dx Codes:** 733.14, 820.0, 820.09, 820.1, 820.10, 820.12, 820.19, 820.20, 820.21, 820.3, 820.30, 820.31, 820.8, 821, 821.00, 821.01, 821.10, 821.11, 821.21, 821.29, 821.3, 821.32, 733.15, 820, 820.00, 820.01, 820.02, 820.03, 820.11, 820.13, 820.2, 820.22, 820.32, 820.9, 821.0, 821.20, 821.22, 821.23, 821.30, 821.31, 821.33, 821.39.  **ICD-10 Dx Codes:** M80.051A, M80.052A, M80.059A, M80.851A, M80.852A, M80.859A, M84.451A, M84.452A, M84.453A, M84.459A, M84.651A, M84.652A, M84.653A, M84.659A, M84.750A, M84.751A, M84.752A, M84.753A, M84.754A, M84.755A, M84.756A, M84.757A, M84.758A, M84.759A, S72.0xxx, S72.1xxx, S72.2xxx, S72.3xxx, S72.4xxx, S72.8xxx, S72.9xxx,  **HCPCS Procedure Code:** 27125, 27130, 27220, 27222, 27226, 27227, 27230, 27238, 27240, 27244, 27248, 27269, 27500, 27506, 27509, 27511, 27513, 27514, 27228, 27232, 27235, 27236, 27245, 27246, 27254, 27267, 27268, 27501, 27502, 27503, 27507, 27508, 27510.  **ICD-10 Procedure Code:** 0QH6xxx, 0QH7xxx, 0QH8xxx, 0QH9xxx, 0QHB0xx, 0QHB3xx, 0QHB4xx, 0QHC0xx, 0QHC3xx, 0QHC4xx, 0QR6xxx, 0QR7xxx, 0QS6xxx, 0QS7xxx, 0QS8xxx, 0QS9xxx, 0QSB0xx, 0QSB3xx, 0QSB4xx, 0QSBXxx, 0QSC0xx, 0QSC3xx, 0QSC4xx.  Incident Humerus fractures, or  **ICD-9 Dx Codes:** 812.00, 812.01, 812.03, 812.1, 812.10, 812.12, 812.2, 812.20, 812.21, 812.3, 812.30, 812.4, 812.40, 812.42, 812.50, 812.52, 812.53, 812.59, 733.11, 812.0, 812.02, 812.09, 812.11, 812.13, 812.19, 812.31, 812.41, 812.43, 812.44, 812.49, 812.5, 812.51, 812.54.  **ICD-10 Dx Codes:** M80.021A, M80.022A, M80.029A, M80.829A, M84.421A, M84.422A, M84.429A, M84.622A, M84.629A, M80.821A, M80.822A, M84.621A, S42.2xxx, S42.3xxx, S42.4xxx, S42.9xxx.  **HCPCS Procedure Codes:** 23500, 23600, 23605, 23616, 23630, 23665, 23670, 23675, 23680, 24505, 24515, 24530, 24546, 24560, 24577, 24579, 24586, 23505, 23515, 23615, 23620, 23625, 24500, 24516, 24535, 24538, 24545, 24565, 24566, 24575, 24576, 24582, 24587.  **ICD-10 Procedure Codes:** 0PHCxxx, 0PHDxxx, 0PHFxxx, 0PHGxxx, 0PSCxxx, 0PSDxxx, 0PSFxxx, 0PSGxxx, 0PBDxxx.  Incident Radius and Ulna fractures  **ICD-9 Dx Codes:** 733.12, 813.0, 813.02, 813.03, 813.04, 813.07, 813.1, 813.12, 813.13, 813.17, 813.18, 813.20, 813.22, 813.30, 813.32, 813.4, 813.40, 813.44, 813.50, 813.51, 813.52, 813.54, 813.8, 813.82, 813.00, 813.01, 813.05, 813.06, 813.08, 813.10, 813.11, 813.14, 813.15, 813.16, 813.2, 813.21, 813.23, 813.3, 813.31, 813.33, 813.41, 813.42, 813.43, 813.5, 813.53, 813.80, 813.81, 813.83, 813.9, 813.90, 813.91, 813.92, 813.93.  **ICD-10 Dx Codes:** M80.031A, M80.032A, M80.039A, M84.432A, M84.431A, M84.433A, M84.434A, M84.439A, M84.631A, M84.632A, M84.633A, M84.634A, M84.639A, M80.831A, M80.832A M80.839A, S52.0xxx, S52.1xxx, S52.2xxx, S52.3xxx, S52.5xxx, S52.6xxx, S52.9xxx.  **HCPCS Procedure Codes**: 24586, 24620, 24650, 24665, 24666, 24675, 24685, 25500, 25505, 25525, 25530, 25535, 25565, 25607, 25651, 25652, 24587, 24635, 24655, 24670, 25515, 25520, 25526, 25545, 25560, 25574, 25575, 25600, 25605, 25606, 25608, 25609, 25611, 25620, 25650.  **ICD-9 Procedure code**: 78.53, 79.12, 79.32, 79.02, 79.22, 79.62.  **ICD-10 Procedure codes:** 0PHHxxx, 0PHJxxx, 0PHKxxx, 0PHLxxx, 0PSHxxx, 0PSJxxx, 0PSKxxx, 0PSLxxx. | Event combination: *Inpatient_Diagnosis code primary position, or *Inpatient_Diagnosis code secondary position, or *Outpatient_Diagnosis code with overlap procedure code event. |
| **Secondary Outcome** | **Definition codes** | **Setting / Position** |
| Dual-energy x-ray absorptiometry (DXA) bone mineral density test | **ICD-9 / ICD-10 Procedure Code**: 88.98, BR07ZZ1, BP49ZZ1, BQ00ZZ1, BP4HZZ1, BP4LZZ1, BP4MZZ1, BP4NZZ1, BP48ZZ1, BP4GZZ1, BQ03ZZ1, BR09ZZ1, BP4PZZ1, BQ01ZZ1, BQ04ZZ1, BR00ZZ1, BR0GZZ1.  **HCPCS Procedure Code:** 76076, 76977, 76075, 76070, G0130, 0508T, 0554T, 0555T, 0556T, 0557T, 77078, 77079, 77080, 77081, 77083, 77085, 77086, 78350, 78351 | Inpatient  Outpatient  Carrier Claims  Any position |
| Osteoporosis medication  (Specific drugs, monoclonal antibody, bisphosphonates, estrogen receptor modulators, PTH analogs, and other medications.) | **NDC Generic Name:**   - ZOLEDRONIC ACID, - ZOLEDRONIC ACID IN MANNITOL AND 0.9 % SODIUM CHLORIDE, - ZOLEDRONIC ACID IN MANNITOL AND WATER FOR INJECTION, - DENOSUMAB - ALENDRONATE SODIUM - ALENDRONATE SODIUM/CHOLECALCIFEROL (VITAMIN D3) - IBANDRONATE SODIUM - PAMIDRONATE DISODIUM - RISEDRONATE SODIUM - RISEDRONATE SODIUM/CALCIUM CARBONATE - ZOLEDRONIC ACID - ZOLEDRONIC ACID IN MANNITOL AND 0.9 % SODIUM CHLORIDE - ZOLEDRONIC ACID IN MANNITOL AND WATER FOR INJECTION - RALOXIFENE HCL - ABALOPARATIDE - TERIPARATIDE - CALCITONIN, SALMON, SYNTHETIC - ETIDRONATE DISODIUM - ROMOSOZUMAB-AQQG - TILUDRONATE DISODIUM   **NDC Brand Name:** Prolia, Reclast  **ICD-9 / ICD-10 Dx Codes**: V07.51, Z79.810  **HCPCS Procedure Code:** J0897, J1740, J2430, J3489, J3110, J0630, J1436, J3111 | Drug  Outpatient  Carrier Claims  Any position |
| Low SES: Dual status code (with Medicaid) | This measure dichotomizes [**Dual Status Code**](https://bwh-dope.aetion.com/measures/details/47448/0/0/basics) to be true for the following values:   - 02 = QMB and full Medicaid coverage, including prescription drugs - 03 = Specified Low-Income Medicare Beneficiary (SLMB)-only - 04 = SLMB and full Medicaid coverage, including prescription drugs - 08 = Other dual eligible (not QMB, SLMB, QWDI, or QI) with full Medicaid coverage, including prescription drugs. | Patient Attribute |

Abbreviations: ICD, International Classification of Disease; HCPCS, Healthcare Common Procedure Coding System; Dx, diagnosis; PTH, parathyroid hormone; NDC, National Drug Code; SES, socioeconomic status; QMB, Qualified Medicare Beneficiary; SLMB, Specified Low-Income Medicare Beneficiary; QWDI, Qualified Disabled Working Individuals; QI, Qualifying Individual.

**Supplemental Table 3.** Complete Patient Demographics

| **Population Characteristics** | **All Patients** | **Asian** | **Black** | **Hispanic** | **Native American** | **White** |
| --- | --- | --- | --- | --- | --- | --- |
| **Number (%) or Mean (SD)** | 159,699 (100%) | 3,479 (2.2%) | 8,850 (5.5%) | 4,486 (2.8%) | 1,177 (0.7%) | 139,098 (87.1%) |
| **Age, yr** | 81.8 (8.0) | 83.1 (7.9) | 80.9 (8.6) | 83.2 (8.3) | 79.0 (7.6) | 81.9 (8.0) |
| **Age categories** |  |  |  |  |  |  |
| 66 - 69 yr | 12,691 (7.9%) | 217 (6.2%) | 1,018 (11.5%) | 326 (7.3%) | 160 (13.6%) | 10,574 (7.6%) |
| 70 - 74 yr | 21,509 (13.5%) | 373 (10.7%) | 1,357 (15.3%) | 508 (11.3%) | 187 (15.9%) | 18,662 (13.4%) |
| 75 - 79 yr | 27,424 (17.2%) | 520 (14.9%) | 1,534 (17.3%) | 622 (13.9%) | 261 (22.2%) | 24,057 (17.3%) |
| 80 - 84 yr | 33,357 (20.9%) | 724 (20.8%) | 1,738 (19.6%) | 806 (18.0%) | 271 (23.0%) | 29,288 (21.1%) |
| ≥ 85 yr | 64,718 (40.5%) | 1,645 (47.3%) | 3,203 (36.2%) | 2,224 (49.6%) | 298 (25.3%) | 56,517 (40.6%) |
| **Female** | 114,067 (71.4%) | 2,552 (73.4%) | 6,395 (72.3%) | 3,207 (71.5%) | 899 (76.4%) | 99,420 (71.5%) |
| **Geographic region** |  |  |  |  |  |  |
| Northeast | 31,295 (19.6%) | 646 (18.6%) | 1,333 (15.1%) | 492 (11.0%) | 23 (2.0%) | 28,210 (20.3%) |
| South | 67,746 (42.4%) | 662 (19.0%) | 5,320 (60.1%) | 2,096 (46.7%) | 374 (31.8%) | 58,610 (42.1%) |
| Midwest | 33,922 (21.2%) | 188 (5.4%) | 1,575 (17.8%) | 256 (5.7%) | 141 (12.0%) | 31,419 (22.6%) |
| West | 26,736 (16.7%) | 1,983 (57.0%) | 622 (7.0%) | 1,642 (36.6%) | 639 (54.3%) | 20,859 (15.0%) |
| **Low SES** | 7,921 (5.0%) | 89 (2.6%) | 637 (7.2%) | 273 (6.1%) | 72 (6.1%) | 6,745 (4.8%) |
| **Smoking** | 62,942 (39.4%) | 643 (18.5%) | 3,472 (39.2%) | 1,191 (26.5%) | 469 (39.8%) | 56,353 (40.5%) |
| **BMI categories** |  |  |  |  |  |  |
| BMI < 25 kg/m^2^ | 17,061 (10.7%) | 564 (16.2%) | 1,316 (14.9%) | 569 (12.7%) | 104 (8.8%) | 14,180 (10.2%) |
| BMI 25 - 29.9 kg/m^2^ | 14,558 (9.1%) | 254 (7.3%) | 739 (8.4%) | 498 (11.1%) | 83 (7.1%) | 12,733 (9.2%) |
| BMI 30 - 39.9 kg/m^2^ | 15,895 (10.0%) | 122 (3.5%) | 729 (8.2%) | 440 (9.8%) | 108 (9.2%) | 14,253 (10.2%) |
| BMI ≥ 40 kg/m^2^ | 9,596 (6.0%) | 57 (1.6%) | 553 (6.2%) | 224 (5.0%) | 60 (5.1%) | 8,568 (6.2%) |
| No information on BMI ; n (%) | 112,347 (70.3%) | 2,613 (75.1%) | 6,037 (68.2%) | 3,056 (68.1%) | 868 (73.7%) | 97,961 (70.4%) |
| **Cohort entry year** |  |  |  |  |  |  |
| 2014 | 37,065 (23.2%) | 750 (21.6%) | 2,133 (24.1%) | 1,077 (24.0%) | 252 (21.4%) | 32,385 (23.3%) |
| 2015 | 35,125 (22.0%) | 702 (20.2%) | 1,921 (21.7%) | 906 (20.2%) | 248 (21.1%) | 30,834 (22.2%) |
| 2016 | 20,223 (12.7%) | 426 (12.2%) | 1,193 (13.5%) | 586 (13.1%) | 160 (13.6%) | 17,562 (12.6%) |
| 2017 | 17,739 (11.1%) | 406 (11.7%) | 1,044 (11.8%) | 544 (12.1%) | 113 (9.6%) | 15,312 (11.0%) |
| 2018 | 17,243 (10.8%) | 432 (12.4%) | 1,013 (11.4%) | 507 (11.3%) | 137 (11.6%) | 14,831 (10.7%) |
| 2019 | 17,043 (10.7%) | 410 (11.8%) | 817 (9.2%) | 481 (10.7%) | 121 (10.3%) | 14,870 (10.7%) |
| 2020 | 15,261 (9.6%) | 353 (10.1%) | 729 (8.2%) | 385 (8.6%) | 146 (12.4%) | 13,304 (9.6%) |
| **Comorbidities** |  |  |  |  |  |  |
| Combined Comorbidity Score | 3.4 (3) | 3.0 (3) | 4.3 (3) | 3.7 (3) | 3.5 (3) | 3.3 (3) |
| Diabetic retinopathy | 20,972 (13.1%) | 502 (14.4%) | 1,221 (13.8%) | 773 (17.2%) | 223 (18.9%) | 17,828 (12.8%) |
| Diabetic neuropathy | 43,701 (27.4%) | 795 (22.9%) | 2,603 (29.4%) | 1,513 (33.7%) | 341 (29.0%) | 37,741 (27.1%) |
| Diabetic nephropathy | 37,479 (23.5%) | 1,084 (31.2%) | 2,864 (32.4%) | 1,386 (30.9%) | 384 (32.6%) | 30,989 (22.3%) |
| Diabetic foot ulcer | 15,505 (9.7%) | 184 (5.3%) | 1,028 (11.6%) | 441 (9.8%) | 136 (11.6%) | 13,498 (9.7%) |
| Cognitive impairment | 5,782 (3.6%) | 82 (2.4%) | 269 (3.0%) | 111 (2.5%) | 30 (2.5%) | 5,189 (3.7%) |
| Heart failure | 70,525 (44.2%) | 1,324 (38.1%) | 4,267 (48.2%) | 1,936 (43.2%) | 485 (41.2%) | 61,510 (44.2%) |
| COPD | 52,079 (32.6%) | 752 (21.6%) | 2,628 (29.7%) | 1,330 (29.6%) | 379 (32.2%) | 46,373 (33.3%) |
| CKD Stages 1 - 2 | 9,777 (6.1%) | 207 (5.9%) | 664 (7.5%) | 346 (7.7%) | 60 (5.1%) | 8,336 (6.0%) |
| CKD Stage 3 | 42,988 (26.9%) | 831 (23.9%) | 2,425 (27.4%) | 1,054 (23.5%) | 294 (25.0%) | 37,732 (27.1%) |
| CKD Stages 4 - 5 | 16,985 (10.6%) | 445 (12.8%) | 1,448 (16.4%) | 575 (12.8%) | 151 (12.8%) | 14,033 (10.1%) |
| End-stage kidney disease* | 11,060 (6.9%) | 465 (13.4%) | 1,639 (18.5%) | 636 (14.2%) | 157 (13.3%) | 7,839 (5.6%) |
| Osteoporosis | 45,728 (28.6%) | 1,322 (38.0%) | 1,953 (22.1%) | 1,512 (33.7%) | 365 (31.0%) | 39,859 (28.7%) |
| History of Fractures | 3,861 (2.4%) | 61 (1.8%) | 175 (2.0%) | 108 (2.4%) | 33 (2.8%) | 3,427 (2.5%) |
| Alcohol use disorder | 5,490 (3.4%) | 60 (1.7%) | 440 (5.0%) | 125 (2.8%) | 88 (7.5%) | 4,682 (3.4%) |
| Hypertension | 153,070 (95.8%) | 3,354 (96.4%) | 8,701 (98.3%) | 4,376 (97.5%) | 1,123 (95.4%) | 133,007 (95.6%) |
| Myocardial infarction | 24,891 (15.6%) | 375 (10.8%) | 1,286 (14.5%) | 501 (11.2%) | 175 (14.9%) | 22,220 (16.0%) |
| Ischemic stroke | 39,093 (24.5%) | 807 (23.2%) | 2,574 (29.1%) | 982 (21.9 %) | 234 (19.9%) | 33,869 (24.3%) |
| Atrial fibrillation | 59,152 (37.0%) | 934 (26.8%) | 2,371 (26.8%) | 1,183 (26.4%) | 280 (23.8%) | 53,636 (38.6%) |
| Hyperlipidemia | 131,639 (82.4%) | 2,928 (84.2%) | 6,964 (78.7%) | 3,647 (81.3%) | 822 (69.8%) | 115,062 (82.7%) |
| Depression | 60,781 (38.1%) | 813 (23.4%) | 2,782 (31.4%) | 1,587 (35.4%) | 387 (32.9%) | 54,435 (39.1%) |
| Liver Disease | 14,941 (9.4%) | 480 (13.8%) | 1,161 (13.1%) | 507 (11.3%) | 151 (12.8%) | 12,296 (8.8%) |
| Cancer | 6,320 (4.0%) | 96 (2.8%) | 331 (3.7%) | 148 (3.3%) | 31 (2.6%) | 5,610 (4.0%) |
| Hyperthyroidism | 3,533 (2.2%) | 100 (2.9%) | 282 (3.2%) | 88 (2.0%) | 28 (2.4%) | 2,970 (2.1%) |
| **Frailty scores** |  |  |  |  |  |  |
| < 0.15 | 2,494 (1.6%) | 97 (2.8%) | 94 (1.1%) | 56 (1.2%) | 20 (1.7%) | 2,148 (1.5%) |
| 0.15 - < 0.25 | 50,667 (31.7%) | 1,373 (39.5%) | 2,204 (24.9%) | 1,207 (26.9%) | 433 (36.8%) | 44,432 (31.9%) |
| 0.25 - < 0.35 | 68,625 (43.0%) | 1,399 (40.2%) | 3,876 (43.8%) | 1,886 (42.0%) | 486 (41.3%) | 59,941 (43.1%) |
| ≥ 0.35 | 37,913 (23.7%) | 610 (17.5%) | 2,676 (30.2%) | 1,337 (29.8%) | 238 (20.2%) | 32,577 (23.4%) |
| **Health system utilization (prior 365 days)** |  |  |  |  |  |  |
| DXA testing | 9,992 (6.3%) | 233 (6.7%) | 356 (4.0%) | 300 (6.7%) | 53 (4.5%) | 8,883 (6.4%) |
| Emergency department visit | 2.30 (3.76) | 1.62 (2.84) | 2.97 (4.43) | 2.42 (3.42) | 3.17 (6.30) | 2.27 (3.72) |
| Number of hospitalizations | 0.88 (1.50) | 0.75 (1.38) | 1.17 (1.84) | 0.95 (1.59) | 1.02 (1.61) | 0.86 (1.47) |
| Internist outpatient visit | 125,704 (78.7%) | 2,902 (83.4%) | 6,504 (73.5%) | 3,464 (77.2%) | 906 (77.0%) | 109,844 (79.0%) |
| **Falls** | 39,856 (25.0%) | 630 (18.1%) | 2,046 (23.1%) | 1,003 (22.4%) | 369 (31.4%) | 35,250 (25.3%) |
| **Medications** |  |  |  |  |  |  |
| Number of unique generic medications | 13.5 (7.0) | 13.8 (7.8) | 13.0 (6.9) | 14.5 (7.6) | 13.5 (7.5) | 13.5 (6.9) |
| Number of brand medications | 2.20 (2.15) | 2.97 (2.90) | 2.26 (2.16) | 2.61 (2.47) | 2.33 (2.21) | 2.16 (2.11) |
| Insulin | 35,712 (22.4%) | 714 (20.5%) | 2,319 (26.2%) | 1,297 (28.9%) | 400 (34.0%) | 30,357 (21.8%) |
| SGLT-2 inhibitors | 1,736 (1.1%) | 69 (2.0%) | 58 (0.7%) | 66 (1.5%) | 6 (0.5%) | 1,486 (1.1%) |
| Thiazolidinediones | 5,563 (3.5%) | 223 (6.4%) | 286 (3.2%) | 236 (5.3%) | 61 (5.2%) | 4,634 (3.3%) |
| Osteoporosis medications** | 15,069 (9.4%) | 741 (21.3%) | 508 (5.7%) | 592 (13.2%) | 116 (9.9%) | 12,784 (9.2%) |
| Oral Corticosteroids | 33,368 (20.9%) | 524 (15.1%) | 1,474 (16.7%) | 690 (15.4%) | 227 (19.3%) | 30,041 (21.6%) |
| Proton pump inhibitors | 62,089 (38.9%) | 1,529 (43.9%) | 3,300 (37.3%) | 2,031 (45.3%) | 418 (35.5%) | 53,800 (38.7%) |
| Antidepressants | 44,603 (27.9%) | 480 (13.8%) | 1,559 (17.6%) | 1,087 (24.2%) | 250 (21.2%) | 40,668 (29.2%) |
| Anxiolytics | 16,260 (10.2%) | 433 (12.4%) | 717 (8.1%) | 460 (10.3%) | 96 (8.2%) | 14,280 (10.3%) |
| Benzodiazepines | 38,730 (24.3%) | 518 (14.9%) | 1,491 (16.8%) | 1,180 (26.3%) | 184 (15.6%) | 34,920 (25.1%) |
| Antiarrhythmics | 10,127 (6.3%) | 183 (5.3%) | 385 (4.4%) | 185 (4.1%) | 41 (3.5%) | 9,199 (6.6%) |
| Beta blockers | 87,802 (55.0%) | 1,769 (50.8%) | 4,850 (54.8%) | 2,273 (50.7%) | 532 (45.2%) | 77,029 (55.4%) |
| Calcium channel blockers | 61,505 (38.5%) | 1,744 (50.1%) | 4,680 (52.9%) | 1,904 (42.4%) | 400 (34.0%) | 51,707 (37.2%) |
| ACE inhibitors and ARBs | 91,811 (57.5%) | 2,221 (63.8%) | 5,087 (57.5%) | 2,824 (63.0%) | 735 (62.4%) | 79,371 (57.1%) |
| Gabapentinoids | 32,951 (20.6%) | 686 (19.7%) | 1,847 (20.9%) | 1,028 (22.9%) | 290 (24.6%) | 28,631 (20.6%) |
| Opioids | 71,854 (45.0%) | 1,087 (31.2%) | 4,035 (45.6%) | 1,897 (42.3%) | 627 (53.3%) | 63,302 (45.5%) |

Abbreviations: SES, socioeconomic status; BMI, body mass index (calculated as weight in kilograms divided by height in meters squared); COPD, chronic obstructive pulmonary disease; CKD, chronic kidney disease; DXA, dual-energy X-ray absorptiometry; SGLT-2, Sodium-Glucose Cotransporter 2; ACE, angiotensin-converting enzyme; ARB, angiotensin receptor blockers. * Includes dialysis, ** Excludes calcium and vitamin D.

**Supplemental Table 4.** Hazard ratio for post-fracture mortality between Asian, Black, Hispanic, and Native American versus White individuals in strata of demographic, socioeconomic, and clinical risk factors with overall interaction p-value for each variable or variable category.

| **Variable** | **Asian** | **Black** | **Hispanic** | **Native** | **White** | **Overall Interaction p-value*** |
| --- | --- | --- | --- | --- | --- | --- |
| **Overall** | 0.78 (0.73-0.83) | 1.26 (1.22-1.31) | 0.91 (0.86-0.96) | 1.06 (0.96-1.18) | REF |  |
| **Age, yr.** |  |  |  |  |  | <0.001 |
| 66 – 69 | 1.19 (0.90-1.57) | 1.42 (1.25-1.61) | 1.14 (0.91-1.45) | 1.28 (0.93-1.77) | REF |  |
| 70 – 74 | 0.83 (0.65-1.05) | 1.43 (1.29-1.58) | 1.00 (0.83-1.21) | 1.14 (0.85-1.54) | REF |  |
| 75 – 79 | 0.87 (0.72-1.05) | 1.41 (1.28-1.54) | 0.94 (0.80-1.12) | 1.17 (0.93-1.47) | REF |  |
| 80 – 84 | 0.70 (0.59-0.82) | 1.30 (1.20-1.41) | 0.99 (0.86-1.13) | 1.03 (0.82-1.29) | REF |  |
| ≥ 85 | 0.76 (0.70-0.83) | 1.14 (1.08-1.20) | 0.85 (0.79-0.92) | 0.97 (0.80-1.17) | REF |  |
| **Sex** |  |  |  |  |  | 0.062 |
| Male | 0.83 (0.74-0.93) | 1.18 (1.11-1.26) | 0.95 (0.86-1.04) | 0.97 (0.79-1.19) | REF |  |
| Female | 0.76 (0.70-0.82) | 1.30 (1.24-1.36) | 0.89 (0.83-0.95) | 1.11 (0.98-1.25) | REF |  |
| **Geographic Region** |  |  |  |  |  | 0.038 |
| Northeast | 0.85 (0.73-0.99) | 1.38 (1.25-1.51) | 0.93 (0.79-1.11) | 1.51 (0.78-2.91) | REF |  |
| Midwest | 0.83 (0.71-0.96) | 1.21 (1.16-1.27) | 0.93 (0.85-1.00) | 1.07 (0.89-1.29) | REF |  |
| South | 1.27 (1.00-1.61) | 1.24 (1.14-1.35) | 0.96 (0.77-1.20) | 1.15 (0.85-1.56) | REF |  |
| West | 0.75 (0.68-0.83) | 1.24 (1.08-1.42) | 0.88 (0.80-0.97) | 1.06 (0.91-1.23) | REF |  |
| **Low SES** | 0.65 (0.43-0.99) | 1.00 (0.88-1.14) | 0.65 (0.52-0.82) | 0.78 (0.51-1.19) | REF | <0.001 |
| **Smoking** | 0.86 (0.75-0.99) | 1.18 (1.11-1.25) | 0.89 (0.80-0.99) | 1.10 (0.93-1.29) | REF | 0.026 |
| **Frailty Score** |  |  |  |  |  | 0.022 |
| <0.15 | 0.82 (0.33-2.01) | 2.26 (1.14-4.46) | 1.07 (0.39-2.91) | 1.58 (0.22-11.4) | REF |  |
| 0.15 – <0.25 | 0.94 (0.83-1.08) | 1.30 (1.18-1.43) | 0.98 (0.85-1.13) | 1.38 (1.11-1.71) | REF |  |
| 0.25 – <0.35 | 0.79 (0.72-0.87) | 1.18 (1.12-1.25) | 0.80 (0.73-0.87) | 1.05 (0.90-1.23) | REF |  |
| ≥ 0.35 | 0.86 (0.76-0.98) | 1.09 (1.02-1.15) | 0.85 (0.78-0.92) | 1.04 (0.86-1.27) | REF |  |
| **Comorbidities** |  |  |  |  |  |  |
| Heart Failure | 0.90 (0.83-0.99) | 1.13 (1.08-1.19) | 0.92 (0.85-0.98) | 1.02 (0.88-1.18) | REF | <0.001 |
| COPD | 0.78 (0.69-0.89) | 1.16 (1.09-1.23) | 0.80 (0.73-0.88) | 0.99 (0.83-1.18) | REF | <0.001 |
| CKD Stage 3 | 0.83 (0.74-0.94) | 1.14 (1.07-1.21) | 0.96 (0.86-1.06) | 0.98 (0.80-1.19) | REF | 0.008 |
| CKD Stage 4-5 | 0.89 (0.77-1.03) | 1.01 (0.93-1.09) | 0.92 (0.81-1.04) | 0.90 (0.69-1.16) | REF | <0.001 |
| ESKD | 0.82 (0.71-0.94) | 0.97 (0.90-1.05) | 0.78 (0.69-0.88) | 0.75 (0.58-0.98) | REF | <0.001 |
| **Insulin user** | 0.87 (0.76-1.00) | 1.15 (1.07-1.23) | 0.85 (0.77-0.94) | 0.87 (0.73-1.05) | REF | 0.005 |

Abbreviations: yr, year; SES, socioeconomic status; BMI, body mass index; COPD, chronic obstructive pulmonary disease; CKD, chronic kidney disease; ESKD, end-stage kidney disease. *Age-stratified analysis adjusted for sex only, **Sex-stratified analysis adjusted for age only; other analyses adjusted for both sex and age.
